# [2-Benzyl-3-(naphthalen-1-yl)-2,3-di­hydro-1,2-oxazole-4,5-di­yl]bis­(phenyl­methanone)

**DOI:** 10.1107/S1600536814003250

**Published:** 2014-02-26

**Authors:** R. Sandhya, M. Sithambaresan, M. R. Prathapachandra Kurup

**Affiliations:** a Department of Applied Chemistry, Cochin University of Science and Technology, Kochi 682 022, India; b Department of Chemistry, Faculty of Science, Eastern University, Sri Lanka, Chenkalady, Sri Lanka

## Abstract

In the title compound, C_34_H_25_NO_3_, the five-membered heterocyclic ring adopts an envelope conformation with the N atom as the flap. The plane through the four basal atoms of this ring makes dihedral angles of 69.78 (13), 53.15 (12) and 86.42 (13)°, respectively, with the benzene rings of the benzyl group and the two phenyl­methanone groups at the 4 and 5 positions, and of 78.60 (11)° with the naphthalenyl system. In the crystal, the mol­ecules are linked through C—H⋯O and C—H⋯π contacts into layers parallel to (101).

## Related literature   

For background to isoxazoline dervatives and their applications, see: Gahlot *et al.* (2003 ▶); Kiss *et al.* (2009 ▶); Norman *et al.* (2007 ▶); Shi *et al.* (2012 ▶); Habeeb *et al.* (2001 ▶). For the synthesis of related compounds, see: Chakraborty *et al.* (2012 ▶). For a related structure, see: Sandhya *et al.* (2013 ▶). For ring puckering analysis, see: Cremer & Pople (1975 ▶).
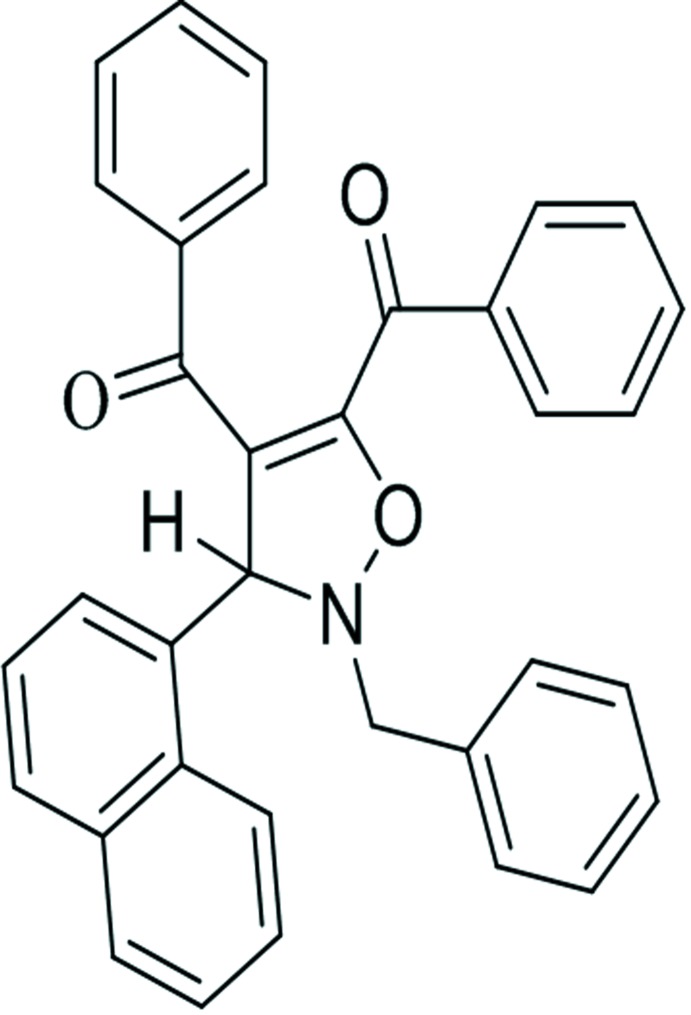


## Experimental   

### 

#### Crystal data  

C_34_H_25_NO_3_
*M* *_r_* = 495.55
Monoclinic, 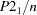

*a* = 14.4105 (10) Å
*b* = 10.9408 (9) Å
*c* = 16.0924 (13) Å
β = 94.235 (4)°
*V* = 2530.2 (3) Å^3^
*Z* = 4
Mo *K*α radiation
μ = 0.08 mm^−1^
*T* = 296 K
0.40 × 0.35 × 0.30 mm

#### Data collection  

Bruker Kappa APEXII CCD area-detector diffractometer
Absorption correction: multi-scan (*SADABS*, Bruker, 2007 ▶) *T*_min_ = 0.968, *T*_max_ = 0.976
12772 measured reflections
5822 independent reflections
3603 reflections with *I* > 2σ(*I*)
*R*_int_ = 0.028

#### Refinement  

*R*[*F* ^2^ > 2σ(*F* ^2^)] = 0.054
*wR*(*F* ^2^) = 0.181
*S* = 1.02
6092 reflections
343 parameters
H-atom parameters constrained
Δρ_max_ = 0.22 e Å^−3^
Δρ_min_ = −0.22 e Å^−3^

### 

Data collection: *APEX2* (Bruker, 2007 ▶); cell refinement: *SAINT* (Bruker, 2007 ▶); data reduction: *SAINT*; program(s) used to solve structure: *SHELXS97* (Sheldrick, 2008 ▶); program(s) used to refine structure: *SHELXL97* (Sheldrick, 2008 ▶); molecular graphics: *ORTEP-3 for Windows* (Farrugia, 2012 ▶) and *DIAMOND* (Brandenburg, 2010 ▶); software used to prepare material for publication: *SHELXL97* and *publCIF* (Westrip, 2010 ▶).

## Supplementary Material

Crystal structure: contains datablock(s) I, Global. DOI: 10.1107/S1600536814003250/yk2103sup1.cif

Structure factors: contains datablock(s) I. DOI: 10.1107/S1600536814003250/yk2103Isup2.hkl

CCDC reference:

Additional supporting information:  crystallographic information; 3D view; checkCIF report

## Figures and Tables

**Table 1:** Hydrogen-bond geometry (Å, °) *Cg*1 is the centroid of the C1–C6 ring.

*D*—H⋯*A*	*D*—H	H⋯*A*	*D*⋯*A*	*D*—H⋯*A*
C5—H5⋯O1^i^	0.93	2.60	3.392 (3)	144
C12—H12⋯O3^ii^	0.93	2.55	3.443 (3)	161
C13—H13⋯*Cg*1^ii^	0.93	2.63	3.523 (3)	161

Symmetry codes: (i) 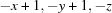; (ii) 
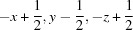
.

**Fig. 1.**
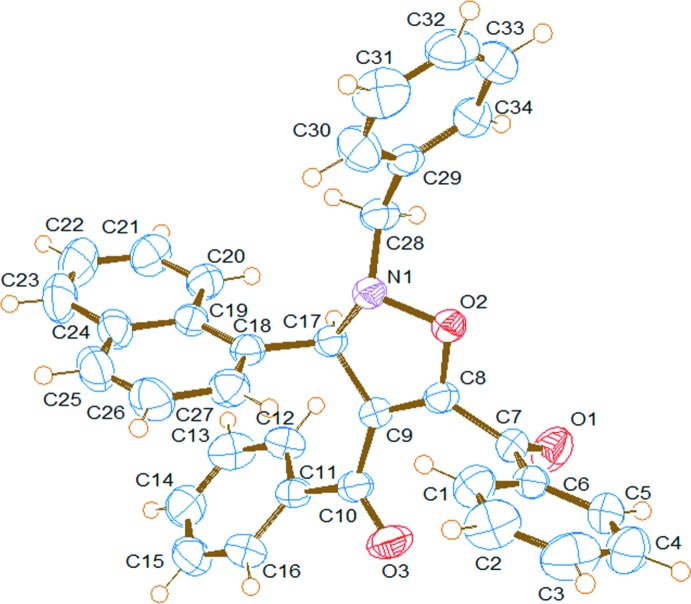
ORTEP view of the title compound drawn with 50% probability displacement ellipsoids for the non-H atoms.

**Fig. 2.**
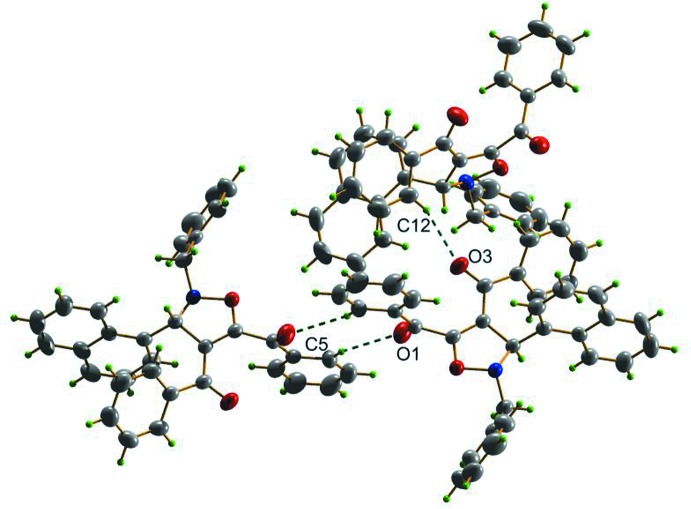
C—H···O intermolecular hydrogen bonding interactions found in the title compound.

**Fig. 3.**
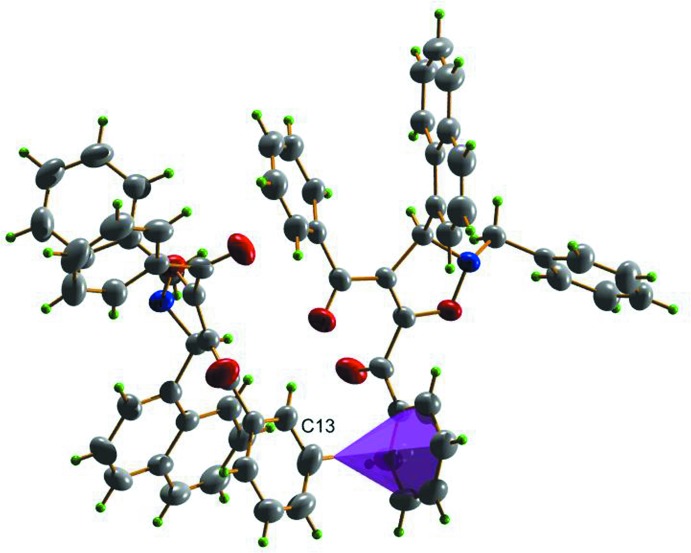
C—H···π interaction found in the title compound.

**Fig. 4.**
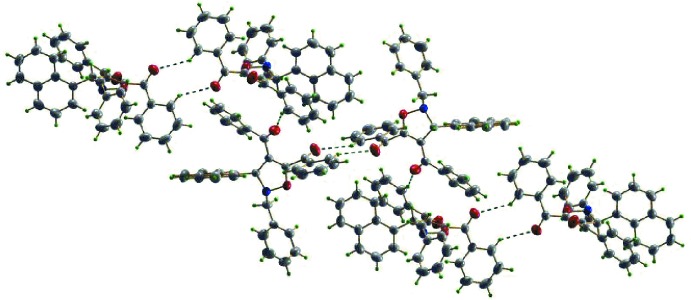
C—H···O intermolecular hydrogen bonding connecting dimers into layers.

**Fig. 5.**
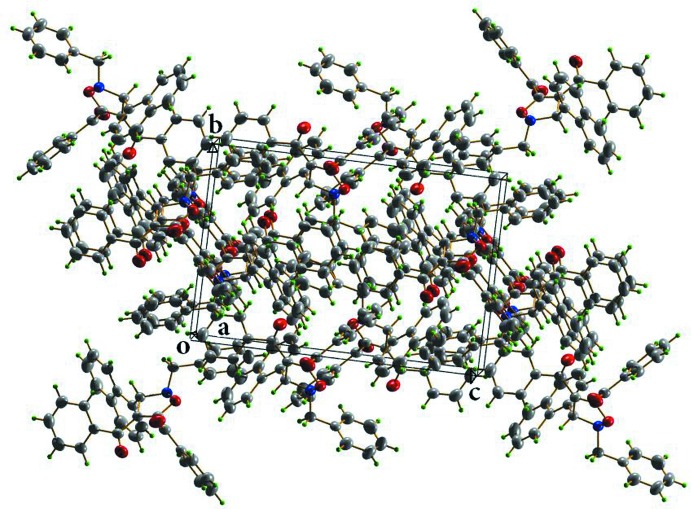
Packing diagram of the compound along the a axis.

## supplementary crystallographic information

### 1. Comment

Isoxazoline derivatives exhibit a wide spectrum of biological activities such as anti-microbial (Gahlot *et al.*, 2003), anti-diabetic (Norman *et al.*, 2007), anti-cancer (Shi *et al.*, 2012) and anti-inflammatory activities (Habeeb *et al.*, 2001). Besides of their pharmacological properties, isoxazolines are also used as intermediates in organic synthesis (Kiss *et al.*, 2009). Herein we report the structure of an isoxazoline derivative synthesized by the 1,3-dipolar cycloaddition reaction of *N*-naphthylidene-*N*-benzylnitrone with dibenzoylacetylene. As a continuous work (Sandhya *et al.*, 2013) on the isoxazoline derivatives, a new compound, (2-benzyl-3-(naphthalen-1-yl)-2,3-dihydroisoxazole-4,5-diyl)bis(phenylmethanone), was prepared and structurally characterized. The ORTEP view of the title compound is shown in Fig. 1.

The compound crystallizes in the monoclinic space group *P*2_1_/n. The dihedral angles between the three aromatic six-membered rings of the benzophenyl (C1–C6 and C11–C16) and benzyl (C29–C34) moieties attached to the heterocyclic ring are 80.27 (12), 60.20 (11) and 63.96 (12)° respectively. The five-membered heterocyclic ring C8/C9/C17/O2/N1 is in an envelope conformation on N1 [φ = 3.9 (3)°] (Cremer & Pople, 1975).

There are two intermolecular C–H···O hydrogen bond interactions (Fig. 2) between the H atoms attached at the C5 & C12 and O1 & O3 atoms of the neighbouring molecule with D···A distances of 3.392 (3) and 3.443 (3) Å respectively. One of the above interaction (C5–H5···O1) forms a centerosymmetric dimer with the adjacent molecule while the other connects such dimers together to build a molecular chain (Fig. 3) in the lattice. An intermolecular C–H···π interaction (Fig. 4) between the H at C13 and the C1-C6 aromatic ring of the neighbouring molecule with H···π distance of 2.63 Å interconnects the molecular chains together. Thus, these intermolecular hydrogen bonding interactions augmented by a C–H···π interaction play a major role to form a supramolecular network in the lattice of the molecular system. Similar intermolecular interactions are found in the compound (2-tert-butyl-3-phenyl-2,3-dihydroisoxazole-4,5-diyl)bis(phenylmethanone) (Sandhya *et al.*, 2013). Fig. 5 shows the packing diagram of the title compound along *a* axis.

### 2. Experimental

The title compound was prepared by adapting a reported procedure (Chakraborty *et al.*, 2012). *N*-naphthylidene-*N*-benzylnitrone, (3 mmol) and dibenzoylacetylene (3 mmol) were added into 15 mL of acetonitrile and stirred for 4 h at room temperature. The reaction was monitored by TLC using EtOAc/hexane (1:5). The solvent was removed under reduced pressure and the product was purified from the crude by column chromatography on silica gel. Colourless crystals suitable for X-ray structure determination were grown from ethanol by slow evaporation (m.p: 134 °C).

### 3. Refinement

All H atoms on C were placed in calculated positions, guided by difference maps, with C–H bond distances of 0.93–0.98 Å. H atoms were assigned *U*_iso_=1.2U_eq_(carrier) or 1.5Ueq (methyl C). Omitted owing to bad disagreement was the reflection (-1 0 1).

### Crystal data

**Table:**

C_34_H_25_NO_3_	*F*(000) = 1040
*M**_r_* = 495.55	*D*_x_ = 1.301 Mg m^−^^3^
Monoclinic, *P*2_1_/*n*	Mo *K*α radiation, λ = 0.71073 Å
Hall symbol: -P 2yn	Cell parameters from 3803 reflections
*a* = 14.4105 (10) Å	θ = 2.3–27.8°
*b* = 10.9408 (9) Å	µ = 0.08 mm^−^^1^
*c* = 16.0924 (13) Å	*T* = 296 K
β = 94.235 (4)°	Block, colourless
*V* = 2530.2 (3) Å^3^	0.40 × 0.35 × 0.30 mm
*Z* = 4	

### Data collection

**Table:**

Bruker Kappa APEXII CCD area-detector diffractometer	5822 independent reflections
Radiation source: fine-focus sealed tube	3603 reflections with *I* > 2σ(*I*)
Graphite monochromator	*R*_int_ = 0.028
ω and φ scan	θ_max_ = 27.5°, θ_min_ = 2.3°
Absorption correction: multi-scan (*SADABS*, Bruker, 2007)	*h* = −16→18
*T*_min_ = 0.968, *T*_max_ = 0.976	*k* = −6→14
12772 measured reflections	*l* = −19→20

### Refinement

**Table:**

Refinement on *F*^2^	Primary atom site location: structure-invariant direct methods
Least-squares matrix: full	Secondary atom site location: difference Fourier map
*R*[*F*^2^ > 2σ(*F*^2^)] = 0.054	Hydrogen site location: inferred from neighbouring sites
*wR*(*F*^2^) = 0.181	H-atom parameters constrained
*S* = 1.02	*w* = 1/[σ^2^(*F*_o_^2^) + (0.0908*P*)^2^ + 0.7195*P*] where *P* = (*F*_o_^2^ + 2*F*_c_^2^)/3
6092 reflections	(Δ/σ)_max_ < 0.001
343 parameters	Δρ_max_ = 0.22 e Å^−^^3^
0 restraints	Δρ_min_ = −0.22 e Å^−^^3^

### Special details

**Table:**

Geometry. All esds (except the esd in the dihedral angle between two l.s. planes) are estimated using the full covariance matrix. The cell esds are taken into account individually in the estimation of esds in distances, angles and torsion angles; correlations between esds in cell parameters are only used when they are defined by crystal symmetry. An approximate (isotropic) treatment of cell esds is used for estimating esds involving l.s. planes.
Refinement. Refinement of F^2^ against ALL reflections. The weighted R-factor wR and goodness of fit S are based on F^2^, conventional R-factors R are based on F, with F set to zero for negative F^2^. The threshold expression of F^2^ > 2sigma(F^2^) is used only for calculating R-factors(gt) etc. and is not relevant to the choice of reflections for refinement. R-factors based on F^2^ are statistically about twice as large as those based on F, and R- factors based on ALL data will be even larger.

### Fractional atomic coordinates and isotropic or equivalent isotropic displacement parameters (Å^2^)

**Table:**

	*x*	*y*	*z*	*U*_iso_*/*U*_eq_	
O1	0.39550 (11)	0.44858 (16)	0.08868 (12)	0.0624 (5)	
O2	0.19673 (10)	0.34089 (13)	0.03919 (9)	0.0432 (4)	
O3	0.26338 (13)	0.62696 (16)	0.20136 (11)	0.0630 (5)	
N1	0.10514 (11)	0.30910 (15)	0.07285 (10)	0.0390 (4)	
C3	0.2856 (2)	0.7599 (3)	−0.12462 (16)	0.0723 (8)	
H3	0.2782	0.8197	−0.1656	0.087*	
C4	0.3717 (2)	0.7075 (3)	−0.10706 (16)	0.0686 (8)	
H4	0.4219	0.7315	−0.1363	0.082*	
C5	0.38332 (17)	0.6192 (2)	−0.04588 (15)	0.0537 (6)	
H5	0.4416	0.5848	−0.0331	0.064*	
C6	0.30779 (14)	0.58189 (18)	−0.00355 (13)	0.0402 (5)	
C7	0.32028 (14)	0.48646 (19)	0.06095 (13)	0.0409 (5)	
C8	0.23384 (13)	0.42734 (18)	0.09116 (12)	0.0381 (4)	
C9	0.19231 (13)	0.44248 (18)	0.16161 (12)	0.0366 (4)	
C17	0.11552 (13)	0.34732 (18)	0.16270 (12)	0.0364 (4)	
H17	0.1356	0.2781	0.1983	0.044*	
C18	0.02157 (13)	0.39606 (18)	0.18652 (12)	0.0384 (4)	
C19	−0.02994 (13)	0.3363 (2)	0.24645 (12)	0.0398 (5)	
C20	−0.00101 (16)	0.2282 (2)	0.28950 (14)	0.0478 (5)	
H20	0.0561	0.1934	0.2798	0.057*	
C21	−0.05523 (18)	0.1743 (3)	0.34471 (15)	0.0602 (6)	
H21	−0.0346	0.1035	0.3723	0.072*	
C22	−0.14162 (19)	0.2239 (3)	0.36060 (16)	0.0702 (8)	
H22	−0.1784	0.1854	0.3979	0.084*	
C1	0.22138 (16)	0.6349 (2)	−0.02256 (15)	0.0523 (6)	
H1	0.1704	0.6096	0.0052	0.063*	
C2	0.2108 (2)	0.7250 (3)	−0.08236 (17)	0.0677 (7)	
H2	0.1532	0.7620	−0.0940	0.081*	
C28	0.09503 (15)	0.17655 (19)	0.06203 (13)	0.0446 (5)	
H28A	0.0495	0.1464	0.0985	0.054*	
H28B	0.1540	0.1372	0.0779	0.054*	
C29	0.06476 (14)	0.14371 (18)	−0.02659 (12)	0.0388 (5)	
C34	0.11531 (16)	0.0652 (2)	−0.07236 (15)	0.0540 (6)	
H34	0.1706	0.0321	−0.0488	0.065*	
C33	0.08423 (19)	0.0350 (3)	−0.15369 (17)	0.0665 (7)	
H33	0.1192	−0.0182	−0.1839	0.080*	
C32	0.0036 (2)	0.0818 (3)	−0.18963 (16)	0.0656 (7)	
H32	−0.0161	0.0619	−0.2443	0.079*	
C31	−0.0483 (2)	0.1589 (3)	−0.14438 (17)	0.0718 (8)	
H31	−0.1041	0.1905	−0.1680	0.086*	
C30	−0.01775 (18)	0.1895 (2)	−0.06364 (16)	0.0586 (6)	
H30	−0.0534	0.2419	−0.0335	0.070*	
C10	0.21642 (14)	0.53967 (19)	0.22180 (13)	0.0413 (5)	
C11	0.18224 (13)	0.53279 (19)	0.30646 (12)	0.0388 (5)	
C16	0.15152 (16)	0.6376 (2)	0.34411 (14)	0.0511 (6)	
H16	0.1521	0.7120	0.3161	0.061*	
C15	0.12022 (18)	0.6322 (3)	0.42265 (16)	0.0612 (7)	
H15	0.0991	0.7026	0.4475	0.073*	
C14	0.12018 (18)	0.5226 (3)	0.46435 (16)	0.0630 (7)	
H14	0.0988	0.5192	0.5174	0.076*	
C13	0.15140 (19)	0.4183 (3)	0.42821 (15)	0.0616 (7)	
H13	0.1521	0.3446	0.4571	0.074*	
C12	0.18182 (16)	0.4228 (2)	0.34900 (13)	0.0481 (5)	
H12	0.2021	0.3519	0.3241	0.058*	
C23	−0.17159 (18)	0.3275 (3)	0.32192 (16)	0.0673 (8)	
H23	−0.2288	0.3603	0.3333	0.081*	
C24	−0.11730 (15)	0.3873 (2)	0.26426 (14)	0.0497 (6)	
C25	−0.14949 (17)	0.4948 (3)	0.22237 (17)	0.0617 (7)	
H25	−0.2066	0.5278	0.2339	0.074*	
C26	−0.09828 (18)	0.5506 (2)	0.16541 (17)	0.0613 (7)	
H26	−0.1197	0.6217	0.1388	0.074*	
C27	−0.01301 (16)	0.4992 (2)	0.14757 (15)	0.0495 (5)	
H27	0.0213	0.5365	0.1079	0.059*	

### Atomic displacement parameters (Å^2^)

**Table:**

	*U*^11^	*U*^22^	*U*^33^	*U*^12^	*U*^13^	*U*^23^
O1	0.0422 (9)	0.0586 (11)	0.0866 (13)	0.0009 (7)	0.0058 (8)	0.0176 (9)
O2	0.0480 (8)	0.0400 (8)	0.0431 (8)	−0.0091 (6)	0.0131 (6)	−0.0046 (6)
O3	0.0778 (12)	0.0517 (10)	0.0607 (10)	−0.0289 (9)	0.0140 (9)	−0.0076 (8)
N1	0.0434 (9)	0.0356 (9)	0.0388 (9)	−0.0078 (7)	0.0088 (7)	−0.0023 (7)
C3	0.104 (2)	0.0635 (18)	0.0487 (14)	−0.0184 (16)	−0.0017 (14)	0.0174 (13)
C4	0.086 (2)	0.0664 (18)	0.0564 (16)	−0.0176 (15)	0.0268 (14)	0.0029 (13)
C5	0.0564 (13)	0.0514 (14)	0.0556 (14)	−0.0056 (11)	0.0204 (11)	−0.0035 (11)
C6	0.0467 (11)	0.0341 (11)	0.0405 (11)	−0.0041 (8)	0.0083 (9)	−0.0034 (9)
C7	0.0389 (10)	0.0365 (11)	0.0485 (12)	−0.0032 (8)	0.0111 (9)	−0.0024 (9)
C8	0.0386 (10)	0.0314 (10)	0.0442 (11)	−0.0030 (8)	0.0037 (8)	0.0012 (8)
C9	0.0358 (10)	0.0336 (10)	0.0404 (10)	−0.0049 (8)	0.0035 (8)	−0.0021 (8)
C17	0.0396 (10)	0.0331 (10)	0.0369 (10)	−0.0041 (8)	0.0040 (8)	−0.0008 (8)
C18	0.0389 (10)	0.0355 (11)	0.0404 (11)	−0.0026 (8)	−0.0001 (8)	−0.0042 (9)
C19	0.0402 (10)	0.0440 (12)	0.0350 (10)	−0.0045 (9)	0.0024 (8)	−0.0094 (9)
C20	0.0496 (12)	0.0472 (13)	0.0465 (12)	−0.0027 (10)	0.0033 (10)	0.0005 (10)
C21	0.0656 (15)	0.0671 (17)	0.0486 (13)	−0.0081 (13)	0.0085 (12)	0.0088 (12)
C22	0.0658 (17)	0.098 (2)	0.0493 (15)	−0.0126 (15)	0.0192 (13)	0.0066 (15)
C1	0.0514 (13)	0.0501 (14)	0.0553 (13)	−0.0036 (10)	0.0037 (10)	0.0066 (11)
C2	0.0729 (17)	0.0639 (18)	0.0640 (16)	−0.0023 (13)	−0.0105 (13)	0.0147 (13)
C28	0.0518 (12)	0.0354 (11)	0.0465 (12)	−0.0080 (9)	0.0027 (9)	0.0000 (9)
C29	0.0430 (11)	0.0315 (10)	0.0422 (11)	−0.0090 (8)	0.0054 (9)	0.0015 (8)
C34	0.0439 (12)	0.0585 (15)	0.0599 (14)	−0.0020 (10)	0.0050 (10)	−0.0097 (12)
C33	0.0683 (17)	0.0738 (19)	0.0587 (15)	−0.0083 (14)	0.0132 (13)	−0.0241 (14)
C32	0.0844 (19)	0.0665 (18)	0.0447 (13)	−0.0212 (15)	−0.0026 (13)	−0.0066 (13)
C31	0.0698 (17)	0.073 (2)	0.0683 (17)	0.0023 (14)	−0.0215 (14)	0.0094 (15)
C30	0.0627 (15)	0.0543 (15)	0.0584 (15)	0.0121 (12)	0.0015 (12)	−0.0016 (12)
C10	0.0419 (11)	0.0340 (11)	0.0475 (12)	−0.0049 (8)	0.0011 (9)	−0.0014 (9)
C11	0.0394 (10)	0.0360 (11)	0.0402 (11)	−0.0039 (8)	−0.0030 (8)	−0.0035 (8)
C16	0.0616 (14)	0.0389 (12)	0.0522 (13)	0.0008 (10)	−0.0001 (11)	−0.0040 (10)
C15	0.0645 (15)	0.0602 (17)	0.0593 (15)	0.0035 (12)	0.0077 (12)	−0.0161 (13)
C14	0.0679 (16)	0.0777 (19)	0.0441 (13)	−0.0106 (14)	0.0084 (11)	−0.0074 (13)
C13	0.0794 (17)	0.0563 (16)	0.0475 (14)	−0.0108 (13)	−0.0069 (12)	0.0084 (12)
C12	0.0613 (14)	0.0392 (12)	0.0425 (12)	−0.0001 (10)	−0.0035 (10)	−0.0020 (9)
C23	0.0542 (14)	0.095 (2)	0.0547 (15)	0.0045 (14)	0.0191 (12)	−0.0079 (15)
C24	0.0451 (12)	0.0604 (15)	0.0439 (12)	0.0026 (10)	0.0055 (9)	−0.0119 (11)
C25	0.0494 (13)	0.0660 (17)	0.0698 (16)	0.0146 (12)	0.0064 (12)	−0.0136 (14)
C26	0.0595 (15)	0.0477 (15)	0.0758 (17)	0.0142 (11)	−0.0016 (13)	0.0008 (13)
C27	0.0504 (12)	0.0413 (12)	0.0566 (13)	0.0013 (10)	0.0019 (10)	0.0037 (10)

### Geometric parameters (Å, º)

**Table:**

O1—C7	1.214 (3)	C28—C29	1.504 (3)
O2—C8	1.347 (2)	C28—H28A	0.9700
O2—N1	1.504 (2)	C28—H28B	0.9700
O3—C10	1.229 (2)	C29—C34	1.375 (3)
N1—C28	1.467 (3)	C29—C30	1.384 (3)
N1—C17	1.502 (2)	C34—C33	1.392 (3)
C3—C2	1.371 (4)	C34—H34	0.9300
C3—C4	1.376 (4)	C33—C32	1.359 (4)
C3—H3	0.9300	C33—H33	0.9300
C4—C5	1.381 (4)	C32—C31	1.372 (4)
C4—H4	0.9300	C32—H32	0.9300
C5—C6	1.387 (3)	C31—C30	1.382 (4)
C5—H5	0.9300	C31—H31	0.9300
C6—C1	1.387 (3)	C30—H30	0.9300
C6—C7	1.474 (3)	C10—C11	1.484 (3)
C7—C8	1.515 (3)	C11—C12	1.385 (3)
C8—C9	1.331 (3)	C11—C16	1.385 (3)
C9—C10	1.463 (3)	C16—C15	1.374 (3)
C9—C17	1.520 (3)	C16—H16	0.9300
C17—C18	1.530 (3)	C15—C14	1.374 (4)
C17—H17	0.9800	C15—H15	0.9300
C18—C27	1.367 (3)	C14—C13	1.372 (4)
C18—C19	1.419 (3)	C14—H14	0.9300
C19—C20	1.418 (3)	C13—C12	1.379 (3)
C19—C24	1.425 (3)	C13—H13	0.9300
C20—C21	1.360 (3)	C12—H12	0.9300
C20—H20	0.9300	C23—C24	1.417 (4)
C21—C22	1.399 (4)	C23—H23	0.9300
C21—H21	0.9300	C24—C25	1.416 (4)
C22—C23	1.349 (4)	C25—C26	1.363 (4)
C22—H22	0.9300	C25—H25	0.9300
C1—C2	1.378 (3)	C26—C27	1.400 (3)
C1—H1	0.9300	C26—H26	0.9300
C2—H2	0.9300	C27—H27	0.9300
			
C8—O2—N1	104.92 (13)	C29—C28—H28B	109.3
C28—N1—C17	113.05 (16)	H28A—C28—H28B	108.0
C28—N1—O2	105.55 (14)	C34—C29—C30	117.9 (2)
C17—N1—O2	104.76 (13)	C34—C29—C28	122.0 (2)
C2—C3—C4	120.8 (3)	C30—C29—C28	120.1 (2)
C2—C3—H3	119.6	C29—C34—C33	120.4 (2)
C4—C3—H3	119.6	C29—C34—H34	119.8
C3—C4—C5	119.9 (2)	C33—C34—H34	119.8
C3—C4—H4	120.1	C32—C33—C34	121.1 (3)
C5—C4—H4	120.1	C32—C33—H33	119.5
C4—C5—C6	119.8 (2)	C34—C33—H33	119.5
C4—C5—H5	120.1	C33—C32—C31	119.2 (2)
C6—C5—H5	120.1	C33—C32—H32	120.4
C5—C6—C1	119.5 (2)	C31—C32—H32	120.4
C5—C6—C7	119.6 (2)	C32—C31—C30	120.0 (3)
C1—C6—C7	120.83 (19)	C32—C31—H31	120.0
O1—C7—C6	124.05 (19)	C30—C31—H31	120.0
O1—C7—C8	117.97 (19)	C31—C30—C29	121.4 (2)
C6—C7—C8	117.89 (17)	C31—C30—H30	119.3
C9—C8—O2	115.69 (17)	C29—C30—H30	119.3
C9—C8—C7	130.71 (18)	O3—C10—C9	119.74 (19)
O2—C8—C7	113.49 (16)	O3—C10—C11	120.77 (19)
C8—C9—C10	123.70 (18)	C9—C10—C11	119.48 (17)
C8—C9—C17	107.34 (17)	C12—C11—C16	119.3 (2)
C10—C9—C17	128.87 (17)	C12—C11—C10	120.78 (19)
N1—C17—C9	101.55 (15)	C16—C11—C10	119.87 (19)
N1—C17—C18	108.13 (15)	C15—C16—C11	120.3 (2)
C9—C17—C18	114.99 (16)	C15—C16—H16	119.9
N1—C17—H17	110.6	C11—C16—H16	119.9
C9—C17—H17	110.6	C14—C15—C16	119.9 (2)
C18—C17—H17	110.6	C14—C15—H15	120.0
C27—C18—C19	120.04 (19)	C16—C15—H15	120.0
C27—C18—C17	118.18 (18)	C13—C14—C15	120.5 (2)
C19—C18—C17	121.78 (18)	C13—C14—H14	119.8
C20—C19—C18	124.51 (19)	C15—C14—H14	119.8
C20—C19—C24	117.42 (19)	C14—C13—C12	119.9 (2)
C18—C19—C24	118.1 (2)	C14—C13—H13	120.0
C21—C20—C19	121.2 (2)	C12—C13—H13	120.0
C21—C20—H20	119.4	C13—C12—C11	120.1 (2)
C19—C20—H20	119.4	C13—C12—H12	120.0
C20—C21—C22	120.9 (3)	C11—C12—H12	120.0
C20—C21—H21	119.6	C22—C23—C24	121.1 (2)
C22—C21—H21	119.6	C22—C23—H23	119.4
C23—C22—C21	120.1 (2)	C24—C23—H23	119.4
C23—C22—H22	120.0	C25—C24—C23	121.2 (2)
C21—C22—H22	120.0	C25—C24—C19	119.5 (2)
C2—C1—C6	120.3 (2)	C23—C24—C19	119.3 (2)
C2—C1—H1	119.9	C26—C25—C24	121.2 (2)
C6—C1—H1	119.9	C26—C25—H25	119.4
C3—C2—C1	119.7 (3)	C24—C25—H25	119.4
C3—C2—H2	120.2	C25—C26—C27	119.0 (2)
C1—C2—H2	120.2	C25—C26—H26	120.5
N1—C28—C29	111.61 (17)	C27—C26—H26	120.5
N1—C28—H28A	109.3	C18—C27—C26	122.2 (2)
C29—C28—H28A	109.3	C18—C27—H27	118.9
N1—C28—H28B	109.3	C26—C27—H27	118.9
			
C8—O2—N1—C28	−141.69 (16)	C6—C1—C2—C3	1.7 (4)
C8—O2—N1—C17	−22.12 (18)	C17—N1—C28—C29	167.93 (16)
C2—C3—C4—C5	−0.4 (4)	O2—N1—C28—C29	−78.14 (19)
C3—C4—C5—C6	1.3 (4)	N1—C28—C29—C34	123.4 (2)
C4—C5—C6—C1	−0.7 (3)	N1—C28—C29—C30	−58.8 (3)
C4—C5—C6—C7	179.2 (2)	C30—C29—C34—C33	0.9 (4)
C5—C6—C7—O1	10.7 (3)	C28—C29—C34—C33	178.7 (2)
C1—C6—C7—O1	−169.4 (2)	C29—C34—C33—C32	−0.1 (4)
C5—C6—C7—C8	−165.92 (19)	C34—C33—C32—C31	−0.9 (4)
C1—C6—C7—C8	14.0 (3)	C33—C32—C31—C30	1.0 (4)
N1—O2—C8—C9	12.1 (2)	C32—C31—C30—C29	−0.2 (4)
N1—O2—C8—C7	−171.20 (15)	C34—C29—C30—C31	−0.8 (4)
O1—C7—C8—C9	78.7 (3)	C28—C29—C30—C31	−178.7 (2)
C6—C7—C8—C9	−104.5 (3)	C8—C9—C10—O3	15.1 (3)
O1—C7—C8—O2	−97.4 (2)	C17—C9—C10—O3	−161.0 (2)
C6—C7—C8—O2	79.4 (2)	C8—C9—C10—C11	−166.10 (19)
O2—C8—C9—C10	−173.65 (18)	C17—C9—C10—C11	17.8 (3)
C7—C8—C9—C10	10.3 (3)	O3—C10—C11—C12	−140.3 (2)
O2—C8—C9—C17	3.2 (2)	C9—C10—C11—C12	40.9 (3)
C7—C8—C9—C17	−172.8 (2)	O3—C10—C11—C16	38.8 (3)
C28—N1—C17—C9	137.38 (16)	C9—C10—C11—C16	−140.0 (2)
O2—N1—C17—C9	22.96 (18)	C12—C11—C16—C15	−0.5 (3)
C28—N1—C17—C18	−101.25 (19)	C10—C11—C16—C15	−179.7 (2)
O2—N1—C17—C18	144.34 (15)	C11—C16—C15—C14	0.6 (4)
C8—C9—C17—N1	−16.8 (2)	C16—C15—C14—C13	0.1 (4)
C10—C9—C17—N1	159.83 (19)	C15—C14—C13—C12	−0.9 (4)
C8—C9—C17—C18	−133.25 (18)	C14—C13—C12—C11	1.0 (4)
C10—C9—C17—C18	43.4 (3)	C16—C11—C12—C13	−0.3 (3)
N1—C17—C18—C27	−64.1 (2)	C10—C11—C12—C13	178.8 (2)
C9—C17—C18—C27	48.5 (3)	C21—C22—C23—C24	0.6 (4)
N1—C17—C18—C19	115.07 (19)	C22—C23—C24—C25	178.7 (3)
C9—C17—C18—C19	−132.28 (19)	C22—C23—C24—C19	0.5 (4)
C27—C18—C19—C20	179.1 (2)	C20—C19—C24—C25	−179.5 (2)
C17—C18—C19—C20	−0.1 (3)	C18—C19—C24—C25	−0.2 (3)
C27—C18—C19—C24	−0.2 (3)	C20—C19—C24—C23	−1.3 (3)
C17—C18—C19—C24	−179.36 (18)	C18—C19—C24—C23	178.1 (2)
C18—C19—C20—C21	−178.4 (2)	C23—C24—C25—C26	−178.4 (2)
C24—C19—C20—C21	0.9 (3)	C19—C24—C25—C26	−0.2 (4)
C19—C20—C21—C22	0.2 (4)	C24—C25—C26—C27	0.9 (4)
C20—C21—C22—C23	−1.0 (4)	C19—C18—C27—C26	0.9 (3)
C5—C6—C1—C2	−0.8 (3)	C17—C18—C27—C26	−179.9 (2)
C7—C6—C1—C2	179.3 (2)	C25—C26—C27—C18	−1.3 (4)
C4—C3—C2—C1	−1.1 (4)		

### Hydrogen-bond geometry (Å, º)

Cg1 is the centroid of the C1–C6 ring.

**Table:**

*D*—H···*A*	*D*—H	H···*A*	*D*···*A*	*D*—H···*A*
C5—H5···O1^i^	0.93	2.60	3.392 (3)	144
C12—H12···O3^ii^	0.93	2.55	3.443 (3)	161
C13—H13···*Cg*1^ii^	0.93	2.63	3.523 (3)	161

Symmetry codes: (i) −*x*+1, −*y*+1, −*z*; (ii) −*x*+1/2, *y*−1/2, −*z*+1/2.
